# Effects of Balance Training on Balance Performance in Healthy Older Adults: A Systematic Review and Meta-analysis

**DOI:** 10.1007/s40279-015-0375-y

**Published:** 2015-09-01

**Authors:** Melanie Lesinski, Tibor Hortobágyi, Thomas Muehlbauer, Albert Gollhofer, Urs Granacher

**Affiliations:** Division of Training and Movement Sciences, Research Focus Cognition Sciences, University of Potsdam, Am Neuen Palais 10, Building 12, 14469 Potsdam, Germany; Centre for Human Movement Sciences, University Medical Centre Groningen, Groningen, The Netherlands; Institute of Sport and Sport Science, Albert-Ludwigs-University of Freiburg, Freiburg, Germany

## Abstract

**Background:**

The effects of balance training (BT) in older adults on proxies of postural control and mobility are well documented in the literature. However, evidence-based dose–response relationships in BT modalities (i.e., training period, training frequency, training volume) have not yet been established in healthy older adults.

**Objectives:**

The objectives of this systematic literature review and meta-analysis are to quantify BT intervention effects and to additionally characterize dose–response relationships of BT modalities (e.g., training period, training frequency) through the analysis of randomized controlled trials (RCTs) that could maximize improvements in balance performance in healthy community-dwelling older adults.

**Data Sources:**

A computerized systematic literature search was performed in the electronic databases PubMed and Web of Science from January 1985 up to January 2015 to capture all articles related to BT in healthy old community-dwelling adults.

**Study Eligibility Criteria:**

A systematic approach was used to evaluate the 345 articles identified for initial review. Only RCTs were included if they investigated BT in healthy community-dwelling adults aged ≥65 years and tested at least one behavioral balance performance outcome (e.g., center of pressure displacements during single-leg stance). In total, 23 studies met the inclusionary criteria for review.

**Study Appraisal and Synthesis Methods:**

Weighted mean standardized mean differences between subjects (SMD_bs_) of the intervention-induced adaptations in balance performance were calculated using a random-effects model and tested for an overall intervention effect relative to passive controls. The included studies were coded for the following criteria: training modalities (i.e., training period, training frequency, training volume) and balance outcomes [static/dynamic steady-state (i.e., maintaining a steady position during standing and walking), proactive balance (i.e., anticipation of a predicted perturbation), reactive balance (i.e., compensation of an unpredicted perturbation) as well as balance test batteries (i.e., combined testing of different balance components as for example the Berg Balance Scale)]. Heterogeneity between studies was assessed using *I*^2^ and Chi^2^-statistics. The methodological quality of each study was tested by means of the Physiotherapy Evidence Database (PEDro) Scale.

**Results:**

Weighted mean SMD_bs_ showed that BT is an effective means to improve static steady-state (mean SMD_bs_ = 0.51), dynamic steady-state (mean SMD_bs_ = 0.44), proactive (mean SMD_bs_ = 1.73), and reactive balance (mean SMD_bs_ = 1.01) as well as the performance in balance test batteries (mean SMD_bs_ = 1.52) in healthy older adults. Our analyses regarding dose–response relationships in BT revealed that a training period of 11–12 weeks (mean SMD_bs_= 1.26), a frequency of three training sessions per week (mean SMD_bs_= 1.20), a total number of 36–40 training sessions (mean SMD_bs_ = 1.39), a duration of a single training session of 31–45 min (mean SMD_bs_ = 1.19), and a total duration of 91–120 min of BT per week (mean SMD_bs_ = 1.93) of the applied training modalities is most effective in improving overall balance performance. However, it has to be noted that effect sizes for the respective training modalities were computed independently (i.e., modality specific). Because of the small number of studies that reported detailed information on training volume (i.e., number of exercises per training session, number of sets and/or repetitions per exercise, duration of single-balance exercises) dose–response relationships were not computed for these parameters.

**Limitations:**

The present findings have to be interpreted with caution because we indirectly compared dose–response relationships across studies using SMD_bs_ and not in a single controlled study as it is difficult to separate the impact of a single training modality (e.g., training frequency) from that of the others. Moreover, the quality of the included studies was rather limited with a mean PEDro score of 5 and the heterogeneity between studies was considerable (i.e., *I*^2^ = 76–92 %).

**Conclusions:**

Our detailed analyses revealed that BT is an effective means to improve proxies of static/dynamic steady-state, proactive, and reactive balance as well as performance in balance test batteries in healthy older adults. Furthermore, we were able to establish effective BT modalities to improve balance performance in healthy older adults. Thus, practitioners and therapists are advised to consult the identified dose–response relationships of this systematic literature review and meta-analysis. However, further research of high methodologic quality is needed to determine (1) dose–response relationships of BT in terms of detailed information on training volume (e.g., number of exercises per training session) and (2) a feasible and effective method to regulate training intensity in BT.

## Key Points

**Table:** 

The present systematic review and meta-analysis quantified dose–response relationships of balance training (BT) modalities (i.e., training period, training frequency, training volume) to maximize improvements in balance performance in healthy adults aged 65 years and older.
Our analyses revealed that an effective BT protocol is characterized by the following independently considered training modalities to improve balance performance in healthy older adults: a training period of 11–12 weeks, a frequency of three sessions per week, a total number of 36–40 training sessions, a duration of 31–45 min of a single training session, and a total duration of 91–120 min of BT per week.
Our study provides preliminary evidence-based guidelines on dose–response relationships for practitioners and therapists to increase the efficacy of their BT protocols and to highlight the necessity of studies that incorporate systematically structured BT programs.

## Introduction

Age-related changes in the sensorimotor and neuromuscular system negatively affect performance in static and dynamic postural control even in healthy older adults [1]. Cross-sectional studies highlight that healthy older adults show larger center of pressure displacements (CoP) and sway velocity in bi- and unipedal quiet stances under different conditions (e.g., eyes opened/closed; stable/unstable surface) compared with young adults [2–4]. Critical markers in postural control have been reported in the literature that are associated with an increased risk of falls. For instance, a standing time of ≤19 s in the modified Romberg Test [5], a habitual gait speed of <1 m/s [6], and a duration of ≥13.5 s to complete the Timed-Up-and-Go Test (TUG) [7], are associated with a two- to threefold increased risk of falls. The short- and long-term effects of serious fall-related injuries, such as mobility limitations, functional decline, and dependent care, significantly reduce quality of life and increase the risk of early death [8, 9].

To mitigate age-related declines in balance performance and prevent falls in old age, a number of studies examined the effects of balance training (BT) over the past years [10, 11]. BT primarily aims at improving postural control by challenging the alignment of the body’s center of gravity with regard to the base of support (i.e., feet) [12]. Even though there is evidence from original work that BT is effective in improving measures of postural control and ultimately fall risk and rate in older adults [11, 13–15], there is a void in the literature regarding the aggregation of study findings from original work. This is usually realized by conducting systematic literature reviews and meta-analyses. With regard to the level of evidence, findings from meta-analyses are categorized on the highest evidence level, whereas results from original work [e.g., randomized controlled trials (RCTs)] are classified lower [16].

In a recently published meta-analysis, dose–response relationships were quantified for BT in healthy young adults [17]. These authors quantified training frequency, period, and volume; however, intensity was not quantified because there is no psychometrically sound measure available to describe balance exercise intensity [18]. Findings from the meta-analysis indicated that training modalities mainly behave in an inversed U-shape, indicating optimal as well as below- and above-threshold training stimuli. Compared with healthy young adults, we hypothesize that older adults’ BT dose–response relationships may show a shift in inverse U-shapes that is modality specific. Differences in training status/fitness level may demand age-specific BT protocols to achieve optimal training effects. The well-established training principle of progressive overload implies that training modalities (e.g., training frequency, training volume) should correspond to the current training status of a given person to avoid overload of the respective biological system [19]. In addition to training status, advanced age with its associated neuromuscular degenerative processes (e.g., decrease in number and size of particularly type II muscle fibers, loss of sensory and motor neurons) seem to have an impact on the temporal pattern of adaptive processes following training in terms of more time needed for adaptive processes [20]. Based on these premises, there is sufficient justification to determine the age-specific dose–response relationships following BT in older adults.

To the best of our knowledge, there is currently no systematic review and meta-analysis that reported the dose–response relationships of BT training modalities in healthy older adults. Therefore, the objectives of this systematic literature review and meta-analysis are to quantify BT intervention effects on balance outcomes (static/dynamic steady-state, proactive balance, reactive balance as well as balance test batteries) and to additionally characterize dose–response relationships of BT modalities (i.e., training period, training frequency, training volume) through the analysis of RCTs that could maximize improvements in balance performance in healthy community-dwelling older adults.

## Methods

### Literature Search

We performed a computerized systematic literature search in PubMed and Web of Science from January 1985 up to January 2015. Because there is no consistent term for training that incorporates balance exercises [21], we referred to an already established Boolean search syntax that was introduced by Lesinski et al. [17]: ((“balance training” OR “neuromuscular training” OR “proprioceptive training” OR “sensorimotor training” OR “instability training” OR “perturbation training”) AND (old* OR aged OR senior* OR elder*) NOT (patient* OR disease OR stroke OR Parkinson OR children OR young* OR youth OR adolescents)). In addition, the following filters were activated: text availability: full text; publication dates: 1985/01/01 to 2015/01/31; species: humans, ages: 65+ years; languages: English, German. Further, we checked the reference lists of each included article and we analyzed relevant review articles [14, 22, 23] in an effort to identify additional suitable studies for inclusion in our analyses.

### Selection Criteria

To be eligible for inclusion, studies had to meet the following criteria and report specific experimental characteristics: (a) participants were healthy older adults with a mean age ≥65 years; (b) the study included a BT protocol comprising static/dynamic postural stabilization exercises, and (c) the study tested at least one behavioral balance outcome (e.g., gait speed). Studies with the following features were excluded: (a) non-randomized design; (b) use of only an active control group; (c) inclusion of only one specific type of BT (e.g., balance-related exergames, water-based training, Tai Chi) or a combined type of BT (e.g., balance and resistance training); (d) used fewer than six sessions (i.e., acute studies); (e) participants’ baseline gait speed was <1.0 m/s (in case of a gait speed test) [6], and (f) unavailability of means and standard deviations in the results or if authors did not reply to our request for data. Based on the defined inclusion and exclusion criteria, two independent reviewers (ML, UG) screened potentially relevant papers by analyzing titles, abstracts, and full texts of respective articles to elucidate their eligibility.

### Coding of Studies

Each study was coded for the following variables: number of participants, sex, and age. We coded BT according to the following training modalities:training period, training frequency, and training volume (i.e., number of training sessions, duration of a single training session, total duration of BT per week, number of exercises per training session, number of sets and/or repetition per exercise, duration of a single BT exercise, e.g., standing time). If BT modalities were not reported in detail, the authors were contacted and missing information was requested. This systematic review will not provide information regarding the influence of training intensity, because to date there is no psychometrically sound measure available to describe balance exercise intensity [18].

According to Shumway-Cook and Woollacott [24], balance control is highly task specific and it has to be separated into different categories: static/dynamic steady-state balance (i.e., maintaining a steady position in sitting, standing, and walking), proactive balance (i.e., anticipation of a predicted disturbance), and reactive balance (i.e., compensation for a disturbance) [25]. In fact, several studies indicated that there are only weak to moderate associations between variables of static/dynamic steady-state, proactive, and reactive balance [26, 27]. With reference to these findings, our analyses focused on different balance outcome categories: (a) static steady-state balance (e.g., CoP displacements during single leg stance), (b) dynamic steady-state balance (e.g., 10-m gait speed test), (c) proactive balance (e.g., Functional-Reach-Test or TUG), (d) reactive balance (e.g., CoP displacements after an unexpected perturbation), and (e) balance test batteries (e.g., Berg Balance Scale). When studies reported multiple variables within one of these outcome categories, only one representative outcome variable was included in the analysis. In the category static steady-state balance, highest priority was given to the single right leg stance with eyes opened. As a proxy for dynamic steady-state balance, gait speed was used. The Functional-Reach-Test was preferably selected as a proxy for proactive balance, and finally for reactive balance, we chose CoP displacements following a perturbation impulse. The Berg Balance Scale was used as the most prominent balance test battery. If a study used other tests, we decided to include those tests in our quantitative analyses that were most similar in terms of their temporal/spatial structure to the ones described above (e.g., tandem walking).

Because of the limited number of studies that examined the different outcome categories (i.e., static/dynamic steady-state balance, proactive balance, reactive balance), we quantified overall BT dose–response relationships. When studies reported multiple outcome categories, the following decision tree was applied that prioritized the importance of the respective test instrument to assess functional capacity: (a) balance test batteries, (b) dynamic steady-state balance, (c) reactive balance, (d) proactive balance, and (e) static steady-state balance. If a study implemented an exercise progression scheme over the training period, the mean number of exercises per training session, sets and/or repetitions per exercise, and duration of balance exercises were calculated.

### Assessment of Methodological Quality and Statistical Analyses

The Physiotherapy Evidence Database (PEDro) Scale was used to assess the methodological quality of all eligible intervention studies. The PEDro Scale rates internal study validity and it rates the presence of statistical replicable information on a scale from 0 to 10 with ≥6 representing a cut-off score for high-quality studies [28].

To verify the effectiveness of BT on a balance outcome measures, we computed the within-subject standardized mean difference [SMD_ws_ = ([mean pre-value − mean post-value]/SD pre-value)] and the between-subject standardized mean difference [SMD_bs_ = ([mean post-value intervention group − mean post-value control group]/pooled variance)]. We adjusted the SMD_bs_ for the respective sample size: $g = \left( {1 - \frac{3}{{4N_{i} - 9}}} \right)$, where *N*_*i*_ is the number of subjects [29, 30]. In addition, included studies were weighted according to the magnitude of the respective standard error using Review Manager version 5.3.4 (Copenhagen: The Nordic Cochrane Centre, The Cochrane Collaboration, 2008). A random-effects meta-analysis model was applied to compute the weighted mean SMD_bs_ in Review Manager version 5.3.4. Depending on the respective outcome measure (i.e., sway path vs. time of single leg stance), SMD_ws_/SMD_bs_ can be negative or positive. To improve readability, we reported positive changes in outcomes (SMD_ws_) and superiority of BT compared with the control (SMD_bs_) with a positive SMD_ws_/SMD_bs_. The calculation of SMD_ws_/ SMD_bs_ allows us to conduct a systematic and quantitative evaluation of the different BT modalities including a large number of studies and it helps to determine whether a difference is of practical concern. According to Cohen [31], effect size values of 0.00 ≤ 0.49 indicate small, of 0.50 ≤ 0.79 indicate medium, and of ≥0.80 indicate large effects.

## Results

### Study Characteristics

**Fig. 1:**
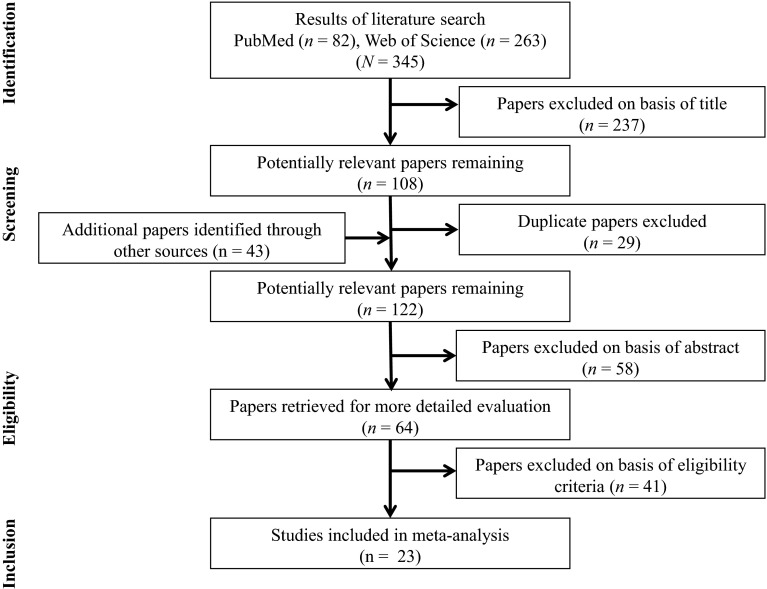
Flow chart illustrating the different phases of the search and study selection

Figure 1 displays a flow chart summarizing the results of the systematic search that identified a total of 345 clinical trials in the electronic databases PubMed and Web of Science. After having added relevant studies from other sources (e.g., reference lists from original work and review articles) and after having screened the articles by title, removed duplicates, and excluded ineligible articles, 23 studies remained and were included in the quantitative analysis.

Table 1 shows the characteristics of the 23 included studies. A total of 1220 subjects participated in the 23 trials and 501 of those subjects received BT. The sample size of the intervention groups ranged from 11 to 75 subjects with a mean age of 66–83 years. The respective training periods of BT interventions ranged from 4 to 15 weeks with a mean value of 9 weeks. The literature search revealed training frequencies ranging from one to seven sessions per week with a mean of three sessions/week and a total of 6–84 training sessions (mean 24 training sessions). Duration of a single training session lasted between 15 and 90 min (mean 56 min) and the total duration of BT per week ranged from 20 to 210 min per week (mean 137 min/week). BT protocols comprised static/dynamic steady-state, proactive, and reactive balance exercises on stable/unstable surfaces (e.g., BOSU^®^ ball, tilt board, trampoline, rocker board, DynaDisc^®^, wobble board, foam mat, balance platform) and balance systems (e.g., Biodex Balance System) with eyes opened or closed. Moreover, many BT protocols contained exercises that were related to activities of daily living, such as obstacle walking. Twelve of 23 studies reported information on progression during training in terms of an increase in level of difficulty of BT. Most studies (*n* = 12) used static steady-state balance tests as the outcome parameter (e.g., center of pressure displacements during unipedal stance) to assess training effects [32–43], seven studies used proxies of dynamic steady-state balance (e.g., gait speed) [34, 35, 38, 42, 44–46], seven studies used proactive balance tests (e.g., Functional-reach-test) [32, 37, 42, 44, 47–49], five studies applied a reactive balance test (e.g., Push-and-release-test) [44, 50–53], and another five studies used a balance test battery (e.g., Berg Balance Scale) [32, 38, 47, 48, 54].

**Table 1:** Studies examining the effects of balance training in healthy older adults

References	Subjects		Balance training modalities					
	*N* (M/F)	Age (years)	*P*	TF	*S*	*D*	*T*	*E*
Arampatzis et al. [50]	38 (13/25)	BT: 67 ± 2 BT + ST: 68 ± 3 CG: 68 ± 3	14	2	28	90	180	N/A
Beling et al. [32]	23 (12/11)	BT: 79 ± 7 CG: 81 ± 5	12	3	36	60	180	5
Bierbaum et al. [51]	38 (13/25)	69 ± 3	14	2	28	90	180	N/A
Franco et al. [54]	32 (7/25)	78 ± 6	3	2	6	30–45	60–90	N/A
Granacher et al. [44]	40 (N/A)	BT: 66 ± 5 CG: 67 ± 4	13	3	36	60	180	N/A
Granacher et al. [45]	20 (6/14)	BT: 72 ± 5 CG: 75 ± 6	6	3	18	60	180	N/A
Gusi et al. [33]	40 (11/29)	76 ± 8	12	2	24	15	30	3
Jacobson et al. [47]	25 (N/A)	63 ± 6	12	3	36	N/A	N/A	N/A
Judge et al. [46]	110 (64/46)	BT: 79 ± 3 ST: 80 ± 4 BT+ ST: 80 ± 4 CG: 81 ± 5	12	3	36	45	135	N/A
Kronhed et al. [34]	30 (14/16)	73 ± 2	9	2	18	60	120	N/A
Leiros-Rodriguez et al. [48]	28 (0/28)	69 ± 3	6	2	12	50	100	12
Maughan et al. [35]	60 (24/36)	BT I: 72 ± 8 BT II: 74 ± 7	6	1	6	20	20	N/A
		CG: 72 ± 8	6	3	18	20	60	N/A
Melzer et al. [36]	66 (17/49)	77 ± 7	12	2	24	N/A	N/A	N/A
Nagai et al. [37]	48 (6/42)	BT: 81 ± 7 CG: 82 ± 6	8	2	16	40	80	8
Pfeifer et al. [38]	33 (4/29)	78 ± 8	4	3	12	60	180	N/A
Piao et al. [39]	30 (16/14)	BT: 68 ± 2 CG: 70 ± 4	8	3	24	60	180	N/A
Rossi et al. [52]	41 (0/41)	BT: 67 ± 2 CG: 68 ± 3	6	3	18	40	120	6
Thiamwong et al. [49]	104 (40/64)	71 ± 8	12	7	84	30	210	6
Weerdesteyn et al. [40]	107 (23/84)	BT: 74 ± 6 CG: 75 ± 7	5	2	10	90	180	N/A
Weerdesteyn et al. [53]	95	BT: 74 ± 6 CG: 75 ± 7	5	2	10	90	180	N/A
Wolf et al. [41]	72 (60/12)	BT: 78 ± 7 Tai Chi: 78 ± 6 CG: 75 ± 5	15	1	15	60	60	N/A
Wolfson et al. [42]	110 (64/46)	79 ± 5	12	3	36	45	135	N/A
Yu et al. [43]	30 (16/14)	BT: 68 ± 2 CG: 70 ± 4	8	3	24	60	180	N/A

References	Subjects		Balance training modalities		Type of balance test	% (pre-post); SMD_ws_	SMD_bs_ (BT vs. control)	
	*N* (M/F)	Age (years)	*S*/*R*	DE				
Arampatzis et al. [50]	38 (13/25)	BT: 67 ± 2 BT + ST: 68 ± 3 CG: 68 ± 3	N/A	N/A	RB (simulation of forward falls; horizontal velocity of the center of mass)	BT: +3.6 % (↑); 0.21	−0.31	
Beling et al. [32]	23 (12/11)	BT:79 ± 7 CG: 81 ± 5	N/A	N/A	sSSB (sensory organization test, composite score)	BT: +20.3 % (↑); 0.96	0.55	
					PB (TUG; time)	BT: −9.3 % (↑); 0.34	0.59	
					TB (BBS; score)	BT: +10.0 % (↑); 0.62	1.76	
Bierbaum et al. [51]	38 (13/25)	69 ± 3	N/A	30–40	RB (gait perturbation test; base of support, mean distance)	BT: +1.9 % (↑); 1.11	−0.17	
Franco et al. [54]	32 (7/25)	78 ± 6	N/A	N/A	TB (BBS; degree)	BT: +10.6 % (↑); 0.59	0.12	
Granacher et al. [44]	40 (N/A)	BT: 66 ± 5 CG: 67 ± 4	4	20	PB (FR; maximal reach distance)	BT: +8.5 % (↑); 0.80	1.00	
					RB (perturbation on Posturomed; sway path)	BT: −60.7 % (↑); 2.19	2.08	
					dSSB (tandem walking; number of successful steps out of 10)	BT: +52.5 % (↑); 0.95	1.47	
Granacher et al. [45]	20 (6/14)	BT: 72 ± 5 CG: 75 ± 6	1/4	20	dSSB (10-m walking test; stride time)	BT: 1.5 % (↑); 0.10	0.83	
Gusi et al. [33]	40 (11/29)	76 ± 8	2–3	10–40	sSSB (20-s bipedal stance on Biodex Balance System; degree)	BT: −48.9 % (↑); 0.96	0.97	
Jacobson et al. [47]	25 (N/A)	63 ± 6	N/A	30–60	PB (TUG; time)	BT: −25.1 % (↑); 0.76	1.33	
					TB (BBS; score)	BT: +51.1 % (↑); 3.77	2.28	
Judge et al. [46]	110 (64/46)	BT: 79 ± 3 ST: 80 ± 4 BT+ ST: 80 ± 4 CG: 81 ± 5	N/A	N/A	dSSB (8-m walking test; usual gait speed)	BT: −2.7 % (↓);−0.14	−0.31	
Kronhed et al. [34]	30 (14/16)	73 ± 2	N/A	N/A	sSSB (right leg stance with eyes opened; time to stand)	BT: −9.5 % (↓); 0.20	0.56	
					dSSB (30-m walking test, usual gait speed)	BT: +11.3 % (↑); 1.13	0.12	
Leiros-Rodriguez et al. [48]	28 (0/28)	69 ± 3	5–60	60	PB (TUG; time)	BT: −39.0 % (↑); 3.30	3.49	
					TB (BBS; score)	BT: 17.9 % (↑); 2.82	2.49	
Maughan et al. [35]	60 (24/36)	BT I: 72 ± 8 BT II: 74 ± 7	N/A	N/A	sSSB (right leg stance with eyes opened; time to stand)	BT: +8.0 % (↑); 0.13	−0.11	
					dSSB (alternating stepping; time)	BT: −5.1 % (↑); 0.75	−0.90	
		CG: 72 ± 8	N/A	N/A	sSSB (right leg stance with eyes opened; time to stand)	BT: +40.0 % (↑); 0.67	0.37	
					dSSB (alternating stepping; time)	BT: −14.6 % (↑); 1.00	−0.09	
Melzer et al. [36]	66 (17/49)	77 ± 7	N/A	N/A	sSSB (30-s bipedal stance with eyes opened; displacements)	BT: −17.2 % (↑); 0.68	1.55	
Nagai et al. [37]	48 (6/42)	BT: 81 ± 7 CG: 82 ± 6	3	>5	sSSB (10-s bipedal stance with eyes opened; postural sway)	BT: −23.5 % (↑); 0.44	0.14	
					PB (FR; maximal reach distance)	BT: +18.3 % (↑); 0.85	1.08	
Pfeifer et al. [38]	33 (4/29)	78 ± 8	N/A	N/A	sSSB (30-s bipedal stance with eyes opened; postural sway path)	BT: −1.3 % (↑); 0.09	−0.45	
					TB (BBS; score)	BT: +14.6 % (↑); 1.64	1.13	
Piao et al. [39]	30 (16/14)	BT: 68 ± 2 CG: 70 ± 4	N/A	N/A	sSSB (30-s bipedal stance with eyes opened; CoP displacements)	BT: −3.5 % (↑); 0.49	0.96	
Rossi et al. [52]	41 (0/41)	BT: 67 ± 2 CG: 68 ± 3	4	60	RB (perturbation test; CoP displacements)	BT: −8.7 % (↑); 2.95	3.26	
Thiamwong et al. [49]	104 (40/64)	71 ± 8	1/20	N/A	PB (FR; maximal reach distance)	BT: +13.0 % (↑); 1.07	1.03	
Weerdesteyn et al. [40]	107 (23/84)	BT: 74 ± 6 CG: 75 ± 7	N/A	N/A	sSSB (single leg stance with eyes opened; time to stand)	BT: +12.2 % (↑); 0.26	0.07	
Weerdesteyn et al. [53]	95	BT: 74 ± 6 CG: 75 ± 7	N/A	N/A	RB (obstacle avoidance; time)	BT: −2.4 % (↑); 0.23	0.31	
Wolf et al. [41]	72 (60/12)	BT: 78 ± 7 Tai Chi: 78 ± 6 CG: 75 ± 5	N/A	10–130	sSSB (single leg stance with eyes opened; postural sway)	BT: −20.6 % (↑); 0.58	−0.08	
							0.72	
Wolfson et al. [42]	110 (64/46)	79 ± 5	N/A	N/A	sSSB (single leg stance with eyes opened; time to stand on a narrow area)	BT: +36.1 % (↑); 1.63	3.19	
					PB (maximal posterior and lateral inclination; inclination angle)	BT: +18.2 % (↑); 2.67	4.68	
					dSSB (8-m walking test, usual gait speed)	BT: +6.3 % (↑); 1.75	1.95	
Yu et al. [43]	30 (16/14)	BT: 68 ± 2 CG: 70 ± 4	N/A	N/A	sSSB (30-s bipedal stance with eyes opened; CoP displacements)	BT: −4.4 % (↑); 0.38	0.42	

*BBS* Berg Balance Scale, *BT* balance training (experimental group), *CG* control group, *CoP* center of pressure, *D* training duration (minutes per training session), *DE* duration of single balance training exercise (seconds), *dSSB* dynamic steady-state balance, *E* number of exercises per training session, *F* female, *FR* Functional-Reach Test, *M* male, *N/A* not available, *P* training period (weeks), *PB* proactive balance, *RB* reactive balance, *S* total number of training sessions, *S/R* number of sets/repetition per exercise, *SMD*_*bs*_ between-subject standardized mean difference, *SMD*_*ws*_ within-subject standardized mean difference, *ST* strength training, *sSSB* static steady state balance, *T* total duration of balance training per week, *TB* balance test battery, *TF* training frequency (times per week), *TUG* Timed-Up-and-Go Test, ↑ indicates performance improvement, ↓ indicates performance decline

### Methodological Quality of the Included Trials

The quality of the included studies can be classified as weak, because 17 out of 23 studies did not reach the predetermined cut-off value of 6 on the PEDro Scale (Table 2) [28]. For all investigated studies, a median PEDro score of 5 (range 3–8) was detected. Additionally, only a few studies reported detailed information regarding the entire BT protocol. Limited and/or incomplete information was specifically reported for training volume (e.g., number of exercises per training session, number of sets per exercise, duration of a single BT exercise) (Table 1).

**Table 2:** Physiotherapy evidence database (PEDro) scores of the reviewed studies

References	Eligi-bility criteria	Rando-mization	Concealed allocation	Similar group baselines	Blinding of all subjects	Blinding of all therapists	Blinding of all assessors	Dropout <15 %	Intention-to-treat method	Statistical between-group comparisons	Point measures and measures of variability	Score
Arampatzis et al. [50]	−	+	−	+	−	−	−	−	−	+	+	4
Beling et al. [32]	+	+	−	+	−	−	−	−	−	+	+	4
Bierbaum et al. [51]	+	+	−	+	−	−	−	−	−	+	+	4
Franco et al. [54]	+	+	−	+	−	−	−	−	−	+	+	4
Granacher et al. [44]	+	+	−	+	−	−	−	−	−	+	+	4
Granacher et al. [45]	+	+	−	+	−	−	−	+	−	+	+	5
Gusi et al. [33]	+	+	+	+	−	−	+	+	+	+	+	8
Jacobson et al. [47]	−	+	−	+	−	−	−	−	−	+	+	4
Judge et al. [46]	+	+	−	+	−	−	+	+	+	+	+	7
Kronhed et al. [34]	+	+	−	−	−	−	−	+	−	+	+	4
Leiros−Rodriguez et al. [48]	+	+	−	+	−	−	−	−	−	+	+	4
Maughan et al. [35]	+	+	−	+	−	−	−	+	+	+	+	6
Melzer et al. [36]	+	+	+	+	−	−	+	+	+	+	+	8
Nagai et al.[37]	+	+	−	+	−	−	−	−	+	+	+	5
Pfeifer et al. [38]	+	+	−	−	−	−	−	−	−	+	+	3
Piao et al. [39]	−	+	−	−	−	−	−	−	−	+	+	3
Rossi et al. [52]	+	+	−	+	−	−	−	+	−	+	+	5
Thiamwong et al. [49]	+	+	−	+	−	−	−	+	−	+	+	5
Weerdesteyn et al. [40]	+	+	−	+	−	−	−	+	+	+	+	6
Weerdesteyn et al. [53]	+	+	−	+	−	−	−	−	−	+	+	4
Wolf et al. [41]	+	+	−	−	−	−	−	−	−	+	+	3
Wolfson et al. [42]	+	+	−	+	−	−	+	+	−	+	+	6
Yu et al. [43]	−	+	−	−	−	−	−	−	−	+	+	3

+ indicates a ‘‘yes’’ score, “−”indicates a ‘‘no’’ score

### Effectiveness of BT

**Fig. 2:**
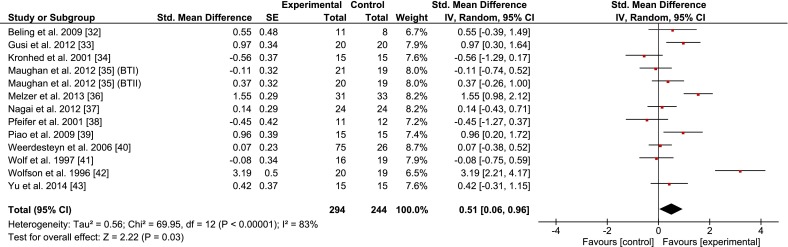
Effects of balance training (experimental) vs. control on measures of static steady-state balance. *CI* confidence interval, *SE* standard error, *Std.* standard, *IV* inverse variance

**Fig. 3:**
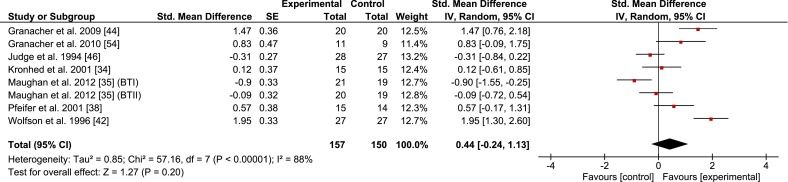
Effects of balance training (experimental) vs. control on measures of dynamic steady-state balance. *CI* confidence interval, *SE* standard error, *Std.* standard, *IV* inverse variance

**Fig. 4:**
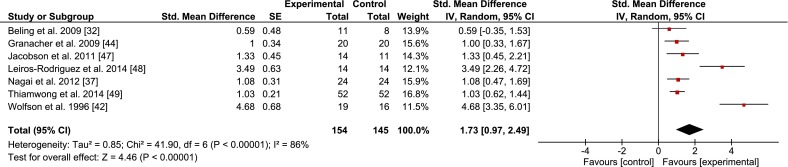
Effects of balance training (experimental) vs. control on measures of proactive balance. *CI* confidence interval, *SE* standard error. *Std.* standard, *IV* inverse variance

**Fig. 5:**
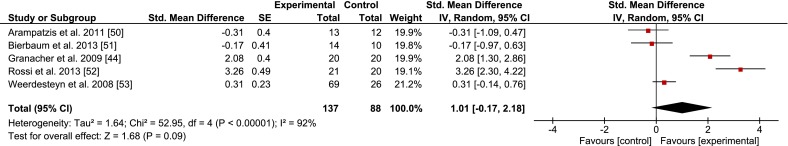
Effects of balance training (experimental) vs. control on measures of reactive balance. *CI* confidence interval, *SE* standard error, *Std.* standard, *IV* inverse variance

**Fig. 6:**
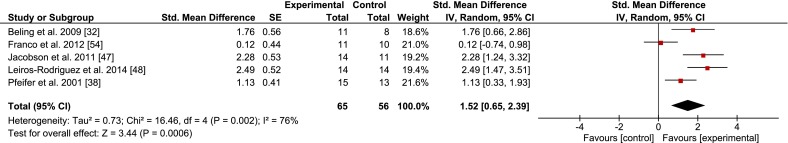
Effects of balance training (experimental) vs. control on performance in balance test batteries, *CI* confidence interval, *SE* standard error, *Std.* standard, *IV* inverse variance

Figures 2, 3, 4, 5, and 6 illustrate the effects of BT vs. a passive control group on proxies of static/dynamic steady-state, proactive, and reactive balance as well as for balance test batteries. Weighted mean SMD_bs_ amounted to 0.51 for measures of static steady-state balance (12 studies; *I*^2^ = 83 %, Chi^2^ = 69.95, *df* = 12, *p* < 0.001), 0.44 for variables of dynamic steady-state balance (7 studies; *I*^2^ = 88 %, Chi^2^ = 57.16, *df* = 7, *p* < 0.001), 1.73 for variables of proactive balance (7 studies; *I*^2^ = 86 %, Chi^2^ = 41.90, *df* = 6, *p* < 0.001), 1.01 for variables of reactive balance (5 studies; *I*^2^ = 92 %, Chi^2^ = 52.95, *df* = 4, *p* < 0.001), and 1.52 for balance test batteries (5 studies; *I*^2^ = 76 %, Chi^2^ = 16.46, *df* = 4, *p* < 0.01), indicating small to large effects.

### Dose–response relationships

**Fig. 7:**
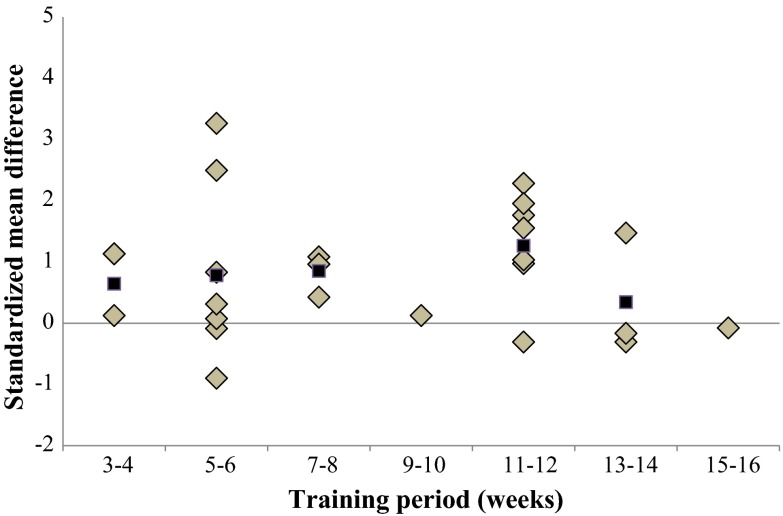
Dose–response relationships of training period on overall balance performance. Each *filled gray diamond* illustrates between-subject standardized mean difference (SMD_bs_) per single study with passive control. *Filled black squares* represent weighted mean SMD_bs_ of all studies

**Fig. 8:**
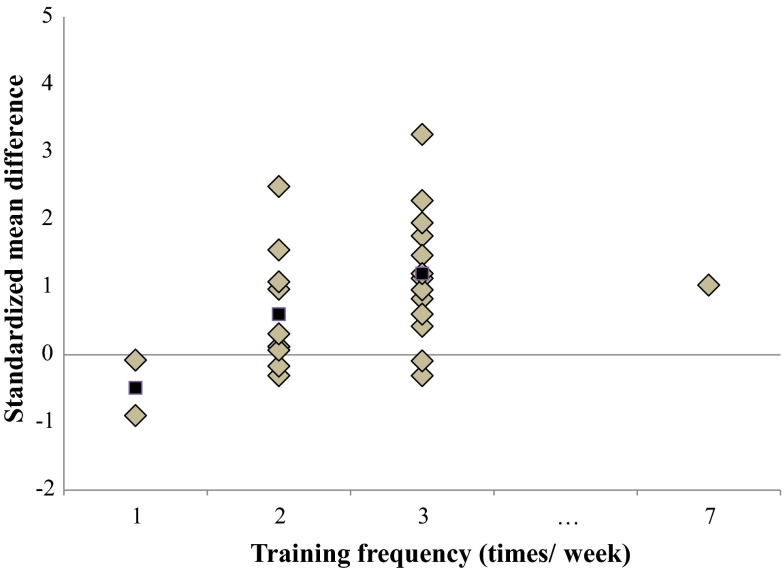
Dose–response relationships of training frequency on overall balance performance. Each *filled gray diamond* illustrates between-subject standardized mean difference (SMD_bs_) per single study with passive control. *Filled black squares* represent weighted mean SMD_bs_ of all studies

**Fig. 9:**
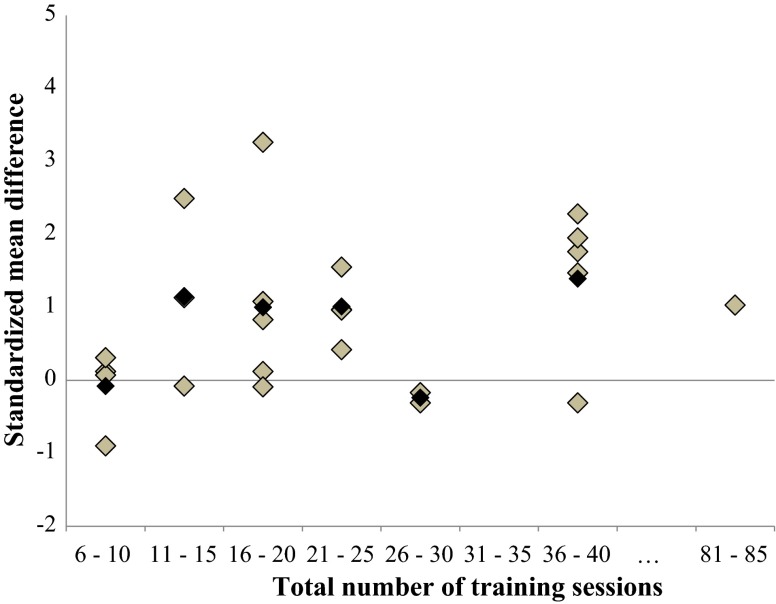
Dose–response relationships of total number of training sessions on overall balance performance. Each *filled gray diamond* illustrates between-subject standardized mean difference (SMD_bs_) per single study with passive control. *Filled black squares* represent weighted mean SMD_bs_ of all studies

**Fig. 10:**
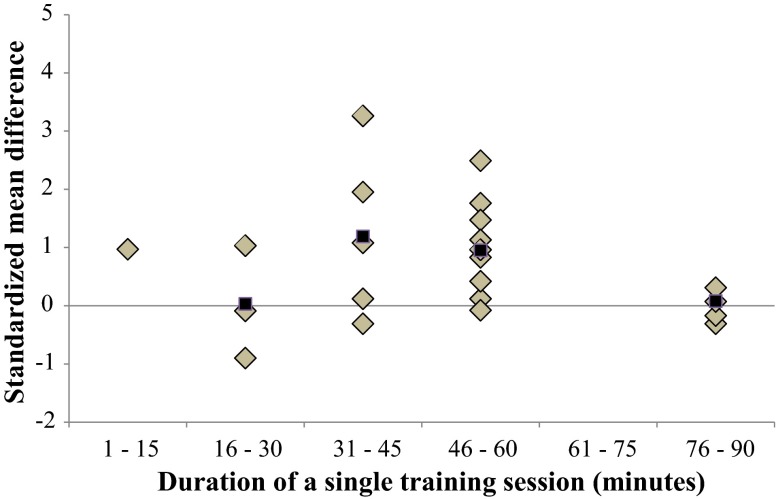
Dose–response relationships of the duration of a single training session on overall balance performance. Each *filled gray diamond* illustrates between-subject standardized mean difference (SMD_bs_) per single study with passive control. *Filled black squares* represent weighted mean SMD_bs_ of all studies

**Fig. 11:**
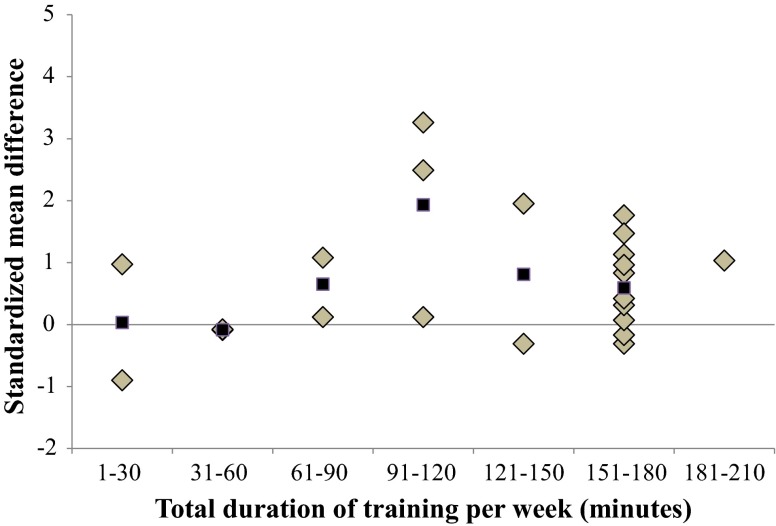
Dose-response relationships of the total duration of balance training per week on overall balance performance. Each *filled gray diamond* illustrates between-subject standardized mean difference (SMD_bs_) per single study with passive control. *Filled black squares* represent mean SMD_bs_ of all studies

Figures 7, 8, 9, 10, and 11 present the overall dose-response relationships (all included studies). Because of the limited number of studies that examined proxies of dynamic steady-state balance, proactive balance, reactive balance, and balance test batteries, specific dose–response relationships were quantified for static steady-state balance only (Table 3). Even though a few authors responded to our inquiries and sent study-specific detailed information on BT modalities, we were not able to quantify dose–response relationships for certain training modalities (i.e., number of exercises per training session, number of sets and/or repetitions per exercise, duration of single BT exercises). Of note, authors did not use a standardized set of BT modalities (e.g., sets, repetitions) to describe the program. Instead, some authors provided time constraints for different BT exercises (e.g., subjects had 10 min time to train different balance exercises with different difficulty level).

**Table 3:** Dose–response relationships for balance training in healthy older adults

Training modalities	Results/most effective dose		
	Healthy older adults		Healthy young adults [17]
	Overall balance	Static steady-state balance	Static steady-state balance
Training period (weeks)	11–12	11–12	11–12
Training frequency (times per week)	3	3	3
Number of training sessions	36–40	36–40	16–19; 36–39^a^
Duration of a single training session (min)	31–45	31–45	11–15^b^
Total duration of BT per week (min)	91–120	121–150 (only one study)	N/A
Number of exercises per training session	N/A	N/A	4
Number of sets/reps per exercise	N/A	N/A	2/N/A
Duration of a single balance exercise (s)	N/A	N/A	21–40

It has to be noted that training modalities were considered independently

*BT* balance training, *N/A* not available, *reps* repetitions

^a^Almost identical effect sizes (1.12 vs. 1.09)

^b^Fourteen out of fifteen studies of BT contained no warm-up and/or cool-down phase and thus were shorter in overall training time than single BT sessions in older adults

#### Training Period

Figure 7 illustrates the overall dose-response relationship for the parameter ‘training period’. Our analyses revealed that a training period of 11–12 weeks produced the largest effects on both overall balance performance (mean SMD_bs_ = 1.26; 23 studies) as well as for more specific measures of static steady-state balance (mean SMD_bs_ = 1.54; 12 studies).

#### Training Frequency

Figure 8 presents the overall dose–response relationship regarding training frequency. A BT frequency of three sessions/week resulted in the largest effects for improving both measures of overall balance performance (mean SMD_bs_ = 1.20; 23 studies) as well as for more specific measures of static steady-state balance (mean SMD_bs_ = 0.81; 12 studies).

#### Training Volume (Number of Training Sessions During the Training Period)

Figure 9 displays the overall dose–response relationship regarding the total number of training sessions. Our findings indicate that a total number of 36–40 training sessions is most effective in improving both overall balance performance (mean SMD_bs_ = 1.39; 23 studies) as well as for more specific measures of static steady-state balance (mean SMD_bs_ = 1.87; 12 studies).

#### Training Volume (Duration of a Single Training Session)

Figure 10 presents the overall dose–response relationship regarding the duration of single training sessions. Our findings revealed that a duration of 31–45 minis most effective to improve overall balance performance (mean SMD_bs_ = 1.19; 21 studies) as well as for more specific measures of static steady-state balance (mean SMD_bs_ = 1.64; 11 studies).

#### Training Volume (Total Duration of Training Per Week)

Figure 11 displays the overall dose–response relationship regarding the total duration of training per week. Our findings indicate that a total duration of 91–120 min of BT per week is most effective in improving overall balance performance (mean SMD_bs_ = 1.93; 21 studies). In terms of improving proxies of static steady-state balance a total duration of 121–150 min (SMD_bs_ = 3.19; one study only) of BT per week produced the largest effects.

## Discussion

This is the first systematic literature review and meta-analysis to examine the overall effects of BT on proxies of balance performance and to characterize and quantify the dose–response relationships of BT modalities (i.e., training period, training frequency, training volume) leading to balance improvements in healthy older adults. Analyses of BT data from 23 RCTs revealed that BT is an effective method to improve healthy older adults’ balance performance. However, the nature of these responses is nearly identical to those reported previously in young adults (Table 3). Against our hypothesis, the results raise the possibility that age does not affect BT parameters known to produce adaptations in static and dynamic measures of balance. We discuss these findings by interpreting the general effects of BT with reference to the already available literature and by taking potential age-specific dose–response relationships into account.

### Effectiveness of Balance Training

A number of reviews and meta-analyses already examined the effects of different fall prevention programs in older adults [11, 14, 55–59] and revealed that among others BT is recommended if the main goal is to reduce risk and rate of falls in older adults [11, 14, 55, 56, 58]. However, there is no systematic review and meta-analysis available that examined the effects of BT on different measures of balance performance (i.e., static/dynamic steady-state balance, proactive balance, reactive balance, balance test batteries). Our analyses showed that BT is effective in improving measures of static/dynamic steady-state, proactive, and reactive balance as well as performance in balance test batteries in healthy old age. Thereby, the effects of BT on measures of static/dynamic steady-state balance are small to medium compared with large effects on proxies of proactive and reactive balance as well as on performance in balance test batteries. Potential ceiling effects may account for the lower effectiveness of BT regarding static/dynamic steady-state balance. Another factor contributing to the small to medium effect sizes is the large difference between the complex temporal and spatial structure of the BT stimuli delivered through the BT programs and the non-specific simple structure of the static balance tests. In terms of dynamic steady-state balance five of seven studies examined habitual gait speed pre- and post-BT. The subjects mean baseline gait speed (1.3 m/s) can be classified as high and is indicative that the included subjects were not mobility limited and had a low risk of falls [60]. Despite the fact that the weighted mean SMD_bs_ of 0.44 was small for proxies of dynamic steady-state balance, the absolute increase in gait speed of 0.07 m/s represents a small but meaningful improvement in gait speed, particularly for healthy older adults [60, 61].

### Dose–response relationships following balance training

The scrutinized studies used a broad range of training periods (4–15 weeks), frequencies (1–7 times/week), number of total training sessions (6–84 training sessions), durations of single training sessions (15–90 min/session), and total durations of BT per week (20–210 min/week). Both the general as well as the specific dose–response relationships for overall balance performance and for measures of static steady-state balance revealed that a training period of 11–12 weeks, a frequency of three sessions per week, a total number of 36–40 training sessions, a duration of a single training session of 31–45 min, and a total duration of 91–120 min of BT per week is most effective to improve balance. Given that only a few included studies reported detailed information on training volume (i.e., the number of exercises per training session, number of sets and/or repetitions per exercise, duration of single BT exercises) as well as examined the effects of BT on measures of dynamic steady-state balance, proactive balance, and reactive balance as well as balance test batteries, we were not able to quantify dose–response relationships for each specific outcome category.

#### Training Period

Our analysis illustrates that BT lasting between 11 and 12 weeks is most effective in enhancing both overall balance performance (mean SMD_bs_ = 1.26; 23 studies) and static steady-state balance (mean SMD_bs_ = 1.54; 12 studies). Figure 7 illustrates that less than 11 weeks of training resulted in lower effects on balance performance. This result is in accordance with Lesinski et al. [17], who quantified the dose–response relationships of BT in young adults (i.e., 18–40 years). Our findings agree with those for young adults in as much as a training period of at least 11–12 weeks is more effective to improve static steady-state balance as compared with shorter training periods (Table 3). Therefore, it seems that there is no age-effect in terms of training period because both meta-analyses observed largest effects when conducting BT for 11–12 weeks. Given that only few studies examined BT periods of more than 12 weeks, this result is preliminary.

A previous review that examined the efficacy of BT to reduce falls [14] concluded that training interventions that involved higher dose of exercise (>50 h) were more effective to reduce falls and recommended at least 2 h of training per week for a training period of 6 months. This might indicate that a BT period of more than 12 weeks could be even more effective in improving overall balance performance.

It is of interest to know whether training-induced adaptations are stable over time or whether they decline during detraining. In this regard, a previous study [62] investigated the effects of static/dynamic BT under single- and dual-task conditions during unipedal stance performance with eyes opened and closed in healthy elderly fallers (*n* = 8; mean age 71 ± 5 years) and non-fallers (*n* = 8; mean age 68 ± 5 years). A 3-month detraining period resulted in a significant decline in unipedal stance performance in fallers and non-fallers. Likewise, Rossi et al. [52] shows that perturbation-based BT for 6 weeks improved neuromuscular responses (e.g., reaction time) following perturbations (i.e., simulation of sudden forward and backward balance loss due to a sliding apparatus) in community-dwelling older women (*n* = 41; mean age 67 ± 3 years). However, the training-induced gains were not stable but declined after 6 weeks of detraining. With reference to the studies of Toulotte et al. [62] and Rossi et al. [52] and the recommendation of Sherrington et al. [14], we advise to conduct BT on a permanent basis to counteract age-related declines in balance performance.

#### Training Frequency

Our analysis revealed that a training frequency of three sessions per week is more effective to improve overall balance performance (mean SMD_bs_ = 1.20; 23 studies) and static steady-state balance (mean SMD_bs_ = 0.81; 12 studies) compared with BT comprising one to two sessions per week. In an intervention study, Maughen et al. [35] examined the specific effects of BT frequency on proxies of static/dynamic steady-state balance in healthy, physically active older adults (*n* = 60; mean age 73 ± 8 years). The authors were able to show that the group that conducted three sessions per week produced larger performance increases after 6 weeks of BT as compared with the group that executed BT once a week. However, the results from this study have to be interpreted with caution because it might be confounded by a higher number of total training sessions (18 vs. 6 training sessions). Still, our findings are confirmed by the recently published systematic review and meta-analysis on dose–response relationships of BT in young healthy adults [17] (Table 3). It appears that there is no age effect in terms of training frequency because both meta-analyses observed largest effects when conducting BT three times per week.

#### Training Volume (Number of Training Sessions)

Concerning the number of training sessions, our analysis revealed that an overall number of 36–40 training sessions produced the largest effects in terms of overall balance performance (mean SMD_bs_= 1.39; 23 studies) and static steady-state balance (mean SMD_bs_ = 1.87; 12 studies). However, given that only one study examined the effects of more than 40 BT sessions, the result is preliminary. Sherrington et al. [14] showed that there are greater benefits from a higher dose of exercise (>50 h) that challenges balance and aims at reducing the risk of falls. Therefore, it might be that BT programs should contains at least 36–40 training sessions but indeed will obtain advantages of more than 40 training sessions in terms of training effects on overall balance performance.

#### Training Volume (Duration of a Single Training Session and Total Duration of Training per Week)

In terms of BT durations, our analyses highlighted that 31–45 min of a single BT session (mean SMD_bs_ = 1.19; 22 studies) and 91–120 min of total BT per week (mean SMD_bs_ = 1.93; 21 studies) seem to be most effective to improve overall balance performance. For improving proxies of static steady-state balance our analysis revealed that 31–45 min of a single BT session (mean SMD_bs_ = 1.64; 11 studies) and 121–150 min (SMD_bs_ = 3.19; one study only) of total BT per week produced the largest effects.

In accordance with the dose–response relationship of BT in young adults [17], there seems to be an inverse U-shaped relation between the effectiveness of BT and the duration of a single training session in old age. However, peak mean SMD_bs_ values shifted to the right, to longer durations (i.e., 31–45 min) in older adults compared with young adults (i.e., 11–15 min). This shift in peak mean SMD_bs_ can most likely be explained by the fact that most BT programs conducted in young adults (particularly in athletes) were either performed immediately before or after the sport-specific training. In older adults, training sessions consisted of BT only, included warm-ups and cool downs, and thus took more time. Taking this into account, the net balance training time appears to be almost similar in healthy older adults compared with young adults. Of note, our detailed analyses revealed that BT durations of more than 60 min produce no additional training effects in older adults. In fact, it seems to be more effective to split the total duration of BT per week (i.e., about 91–120 min) into more (i.e., three or more per week) and shorter (i.e., about 31–45 min) single training sessions, instead of longer single training sessions (i.e., ≥60 min) that are conducted one–two times per week only.

Given that only a few studies reported the number of exercises per training session, the number of sets and/or repetitions per exercise, and the duration of single-balance exercises, dose–response relationships were not computed for these training modalities. In addition, there is no methodological sound approach available in the literature on how to properly assess intensity during BT relative to the individual’s balance ability [18]. Therefore, at this point, it is impossible to establish evidence-based guidelines for all BT modalities in healthy older adults (aged ≥65 years). However, with reference to the best practice recommendations of Sherrington et al. [14], it is possible to present qualitative recommendations on training intensity during BT. Sherrington and colleagues propagate that if the goal is to improve balance and to prevent risk of falling in older adults, moderate to high challenging balance exercises should be conducted in a sufficient dose (at least 50 h, this equate to around 2 h per week for 6 months). Furthermore, if the main aim is the prevention of falls in old age, practitioners should refer patients for the management of other risk factors where appropriate [14]. Falls have multiple interacting predisposing and precipitating causes [55]. Rubenstein [55] listed the important individual risk factor for falls according to 16 controlled studies and deduced the following order of priority: muscle weakness, balance deficit, gait deficit, visual deficit, mobility limitation, cognitive impairment, impaired functional status, and postural hypotension. Therefore, other intervention programs should be included in fall-preventive exercise program (e.g., strength or power training) to target a number of intrinsic fall-risk factors [55].

### Limitations

A limitation of this systematic review is the poor methodological quality of the included studies. Only 6 out of 23 studies were classified as high quality according to the PEDro Scale (PEDro score ≥6). In addition, many studies failed to report data necessary for computing SMD. Thus, future studies should report pre and post means and standard deviations for the investigated balance parameters. Moreover, further research of high methodological quality is needed to determine dose–response relationships of BT for specific training modalities such as training volume (i.e., number of exercises per training session, number of sets and/or repetitions per exercise, duration of a single balance exercise) in healthy older adults and to develop a feasible and effective method to regulate training intensity during BT. In addition, given that it is difficult to separate the impact of each training modality from that of others, that the heterogeneity between studies was considerable (i.e., *I*^2^ = 76–92 %) and that we were not able to take the grade of instability/training intensity that has been trained into account, the present findings are preliminary and have to be interpreted with caution. Further, the highlighted comparisons of dose–response relationships in old vs. young adults are limited because we indirectly compared age-specific dose–response relationships across studies using SMD_bs_ and not in a single controlled study. Finally, findings from this meta-analysis do not allow conclusions with regard to fall-prevention. In other words, our detailed analyses revealed effective BT modalities to improve overall balance performance as well as more specific measures of static steady-state balance. It is unclear how these performance enhancements translate into reduced fall rates.

## Conclusions

Unlike for endurance and resistance training, there are currently no evidence-based recommendations for effective BT protocols (i.e., optimal training modalities) in healthy older adults (aged ≥65 years) available. Therefore, it is not surprising that the identified BT studies in older adults were heterogeneous with regard to the respective training modalities. To provide practitioners and therapists with evidence-based guidelines on effective BT protocols, we investigated the dose–response relationships of BT in healthy older adults. Our analyses revealed that a number of BT modalities (i.e., training period, training frequency, training volume) contribute to the improvements in measures of static/dynamic steady-state, proactive, and reactive balance as well as in the performance of balance test batteries in healthy older adults. An effective BT protocol for healthy older adults is characterized by a training period of 11–12 weeks, a training frequency of three sessions per week, a total number of 36–40 training sessions, a duration of 31–45 min of a single training session, and a total duration of 91–120 min of BT per week. When comparing our findings with those that were recently published in young healthy adults, it seems plausible to argue that almost the same BT protocols are effective in healthy young and older adults, in other words there appears to be no age effect. Given that only a few studies reported detailed information on the number of exercises per training session, the sets and/or repetitions per exercise, and the duration of single exercises dose–response relationships could not be drawn for these parameters. Hence, further research is necessary to prove and specify preliminary dose–response relationships of BT in healthy older adults. In addition, it would be interesting to find out in future studies whether dose–response relationships are significantly different in BT as compared with resistance and endurance training in healthy older adults.
